# Outcomes of acetaminophen infusion on visual analogue scale with varying pain intensity during labour, A randomized controlled trial

**DOI:** 10.12669/pjms.40.10.8425

**Published:** 2024-11

**Authors:** Wajeha Najeeb, Naima Komal, Mudassar Noor, Muhammad Alamgir Khan, Abeera Chaudry

**Affiliations:** 1 Wajeha Najeeb, MBBS. Pakistan Institute of Medical Sciences, Islamabad, Pakistan; 2 Naima Komal, MBBS. Resident Gynaecology and Obstetrics, Pak Emirates Military Hospital, National University of Medical Sciences, Rawalpindi, Pakistan; 3 Mudassar Noor, MPhil, PhD. Associate Professor, Department of Pharmacology, Army Medical College, National University of Medical Sciences, Rawalpindi, Pakistan; 4 Muhammad Alamgir Khan, FCPS, MCPS-HPE. Professor, Department of Physiology, Army Medical College, National University of Medical Sciences, Rawalpindi, Pakistan; 5 Abeera Chaudry, FCPS, FRCS, MRCOG, FRCOG, MCPS-HPE. Professor, Department of Gynaecology and Obstetrics, Pak Emirates Military Hospital, National University of Medical Sciences, Rawalpindi, Pakistan

**Keywords:** Acetaminophen, Visual analogue scales, Labour analgesia

## Abstract

**Objective:**

Acetaminophen (Paracetamol) is the most widely used analgesic. We aimed to determine its efficacy in labour with varying pain intensity, to make labour less painful for women.

**Methods:**

This randomized controlled trial was conducted on primigravida’s in their active phase of first stage of labour at Pharmacology department of Army Medical College, National University of Medical Sciences in collaboration with Gynecology department of Pak Emirates Military hospital, Rawalpindi. It was a registered trial with trial number of IRCT20220308054220N1. Duration of study was from May 2022 to October 2022. Patients were divided into two groups, Group-I received two doses of I/V 100 ml normal saline whereas Group-II received I/V two doses of 1000 mg acetaminophen in 100 ml normal saline. Calculated sample size was 130, 65 in each group. Visual analogue scale was used as a tool for data measurement. Data was analysed using split plot anova test.

**Results:**

Mean VAS in acetaminophen infusion group was found to be less than placebo for 1^st^ dose, but this effect was apparent only one hour after 2^nd^ dose intervention. The difference in means between groups was statistically significant only for 1^st^ dose with p-value of 0.003 (for second dose p-value 0.21). In acetaminophen infusion group, for both doses mean VAS decreased as an immediate effect of drug, but then it increased with time. The difference in VAS between intervals had p-value of <0.001 for 1^st^ dose and 0.005 for 2^nd^ dose.

**Conclusion:**

Acetaminophen is an effective non opioid labour analgesic in active phase of first stage of labour.

## INTRODUCTION

International Association for the Study of Pain (IASP) in its latest modified definition elaborates pain as “An unpleasant sensory and emotional experience associated with or resembling that associated with, actual or potential tissue damage”.^1^ Labour pain is not a pathological process but rather a physiological phenomenon.^2^ It has two components, visceral pain, in the early first stage and second stage of labour, and somatic pain, in the late first stage and second stage of labour.^3^

It is proven scientifically that intensity and severity of labour pain surmount the most excruciating pain experiences one can ever think of.^2^,^4^ Intrapartum analgesia is desired by most of the parturients all over the world.^5^ Researchers are trying for years to devise techniques and drugs to diminish labor pains with minimum side effects.^6^ This resulted in advent of various pharmacological as well as non-pharmacological labour analgesics. Pharmacological approaches like epidural analgesics and opioids are being used effectively but these are not devoid of side effects.^2^ Acetaminophen is a widely prescribed analgesic and antipyretic famous for being a well-tolerated and safest medication.^7^ Contrary to other Non-Steroidal Anti-inflammatory drugs (NSAIDs), it has no anti-inflammatory activity and its analgesic effect is caused by both central and peripheral action.^8^

In our low resource settings, there is almost no understanding of intrapartum analgesia.^4^ Bulk of obstetric services are mainly provided by lady health workers, nurses, and non-specialized doctors due to deficient healthcare system. In addition, available obstetric analgesics need expensive equipment, constant monitoring facilities, and trained anaesthesiologists, all of which are not available in under privileged regions of Pakistan.^9^ To provide a safe alternative for labour analgesia to primigravida of developing countries, the need to establish safety and efficacy of Acetaminophen like drugs has increased. This analgesic is easily accessible, economical, has least adverse effects and requires no special administration techniques. These characteristics augment its probability to be an impactful routine labour analgesic.

The current study had three objectives. First was to determine the efficacy of acetaminophen infusion as labour analgesic in primigravida. Second was to evaluate analgesic effect of acetaminophen infusion on repeating the dose after four hours. Thirdly, we aimed to assess pattern of analgesic effect of acetaminophen infusion with time and progressively increasing severity of labour pains. This study was planned with rationale to inform clinicians about the efficacy of acetaminophen in labour and to promote making use of this labour analgesic more common in our country.

## METHODS

It was designed as a placebo double blinded randomized controlled trial conducted at Pharmacology Department, Army Medical College, National University of Medical Sciences, in collaboration with Gynecology Department of Pak Emirates Military Hospital, Rawalpindi.

### Ethical Approval:

The study protocol was approved by ethical review committee of institute (Reference number: ERC/ID/196 dated 17 March 2022), and registered in Iranian Registry of Clinical Trials (IRCT) with trial number IRCT20220308054220N1.

**Fig.1:**
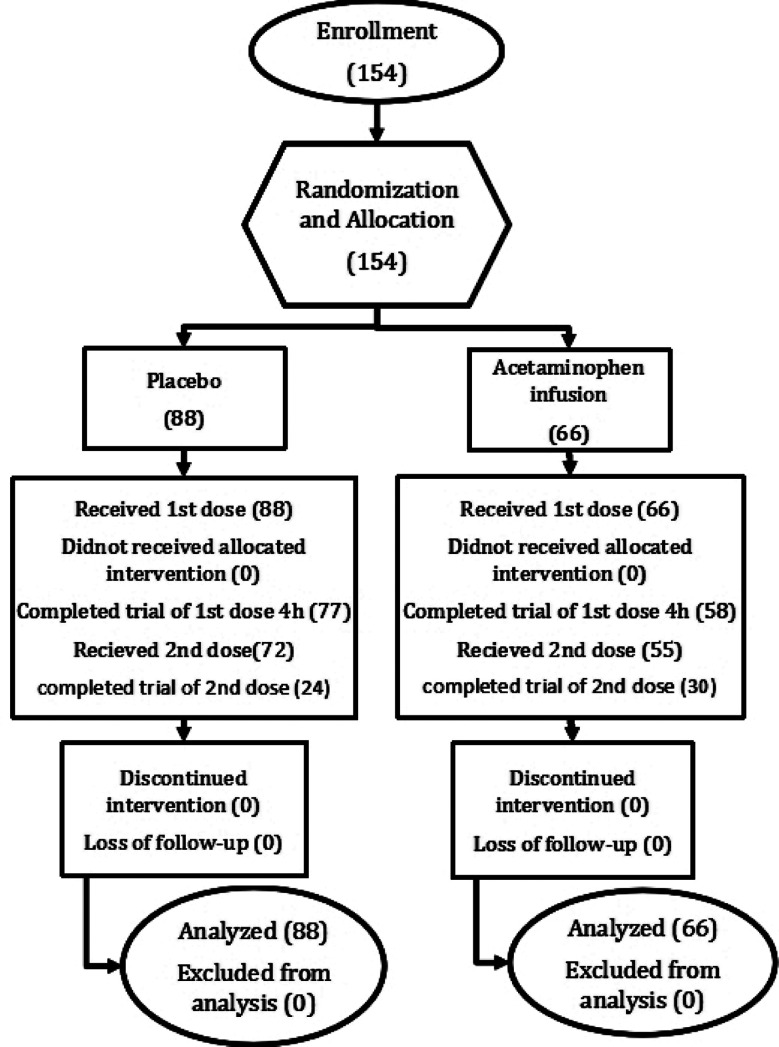
Consort flow diagram.

Duration of study was six months from May 2022 to October 2022. Sample size, was calculated by using G power software. Parent article^10^ mean difference of Visual Analogue Score (VAS) between acetaminophen infusion and placebo group was 0.83 at 60 min post intervention interval with p-value of 0.001. The effect size of parent study was 0.90, calculated by Cohen’s D formula. Two tailed hypothesis was used with significance level of 0.05 and 95% power. Using aforementioned parameters, it was seen that 34 participants in each group with total of 68 participants would be required to obtain statistically significant results. As labour is an unanticipated natural phenomenon, considering 85% dropout rate, we increased sample size to 65 in each group making total of 130 participants. Consort flow diagram of this study is given below (Fig.1).

Educational status of our population ranged from illiterate to literate. Convenient sampling technique was used for enrolment. Women who gave consent were randomized by computer generated codes. Double blinding was achieved by selecting acetaminophen infusion bottles of same shape and size as of 100ml normal saline. Bottles of both acetaminophen infusion and normal saline were also tagged with computer generated codes. Patients and obstetricians attending were both made unaware of the type of drug administered. VAS of 10cm was used as a tool for assessment of pain intensity. Due to subjective perception of pain, visual analogue scale is mostly in use by researchers to assess the effect of painkillers on the severity of pain.^11^

Group-I served as a control group called placebo group and received 100 ml I/V normal saline. Group-II called acetaminophen infusion group acted as an intervention group and included women who were treated with acetaminophen infusion (1000mg in 100ml normal saline). Intervention was done in primigravida in their active phase of first stage of labour with 3-5cm cervical dilatation. Drug was repeated in the same dose after four hours to the parturients who did not deliver during this interval in both groups. VAS was measured before and after at 15 minutes, 30 minutes, one hour, two hours, three hours and four hours of intervention. The same sequence of pain assessment was followed after second dose. Blood pressure, pulse and Body Mass Index (BMI) of participants were recorded before intervention.

### Inclusion criteria:

It included primigravida of reproductive age group at term with uncomplicated and spontaneous or induced labour onset, singleton pregnancy, cephalic presentation and 3-5 cm cervical dilatation.

### Exclusion criteria:

It included malpresentation, multiparous, scarred uterus (post myomectomy, post caesarean), preterm labour, antepartum haemorrhage, drug allergy or hypersensitivity, fetal distress, intrauterine death, refusal by parturient, alcohol or drug abuse history, sepsis, abnormal coagulation profile, obstetric complications (e.g., premature rupture of amniotic membranes) and clinical evidence of cephalopelvic disproportion.

The participants were briefed regarding the benefits and expected outcomes of the study prior to enrolment. They were also informed that there would be no compensation or reward in exchange for their involvement and they would be free to withdraw participation at any moment. Confidentiality of data was ensured. Parturients who experienced any adverse effect or unlikely post intervention response were given medications and health care facility free of cost.

### Statistical analysis:

Data was analysed by SPSS version 25. For quantitative variables, means and standard deviations were calculated. Demographic variables were compared between two groups with the help of Mann-Whitney U-test. Visual analogue scale between subjects and within subject analysis was done by Split plot ANOVA for both doses separately. The *p*-value of ≤0.05 was considered statistically significantly.

## RESULTS

There were 154 participants enrolled considering inclusion and exclusion criteria. Out of these 88 were allocated to placebo group and 66 to acetaminophen infusion group. All of them were primigravidae with 3-5 cm cervical dilatation. Demographic variables including age of parturient, gestational age, BMI, and vitals recorded before administration of drugs, were comparable between groups as explained in Table-I.

**Table-I:** Comparison of demographic and clinical data between groups.

Variables	Groups	Mean ±SD	Sig.
Age (years)	Placebo	25.80 ±3.61	0.45
	Acetaminophen infusion	25.29 ±2.84	
BMI (kg/m^2^)	Placebo	27.03 ±4.10	0.57
	Acetaminophen infusion	26.52 ±3.64	
Gestational age (weeks)	Placebo	38.68 ±1.03	0.73
	Acetaminophen infusion	38.56 ±1.60	
Pulse (beats/min)	Placebo	83.91 ± 3.19	0.04*
	Acetaminophen infusion	82.79 ± 3.64	
Systolic Blood Pressure (mmHg)	Placebo	118.35 ± 6.23	0.17
	Acetaminophen infusion	117± 7.50	
Diastolic Blood Pressure (mmHg)	Placebo	75.90 ± 6.09	0.47
	Acetaminophen infusion	74.94± 5.29	

* . Significant level ≤.05.

History of bleeding in pregnancy was positive in 10% of the participants in placebo group and 4.5% in acetaminophen infusion group, with statistically insignificant *p*-value of 0.236. History of Gestational diabetes mellitus was also statistically insignificant between groups (4.5% in placebo group vs. 6% in acetaminophen infusion group, *p*-value 0.328). Participants enrolled with the history of pregnancy induced hypertension were 6.8% in placebo group and 1.5% in acetaminophen infusion group, with statistically insignificant results (*p*-value 0.240). Per vaginal labour induction trial was given to women for spontaneous vaginal delivery in 41 participants of placebo group and 25 participants of acetaminophen infusion group. This finding between both groups was also comparable with statistical insignificance (*p*-value of 0.325).

**Fig.2:**
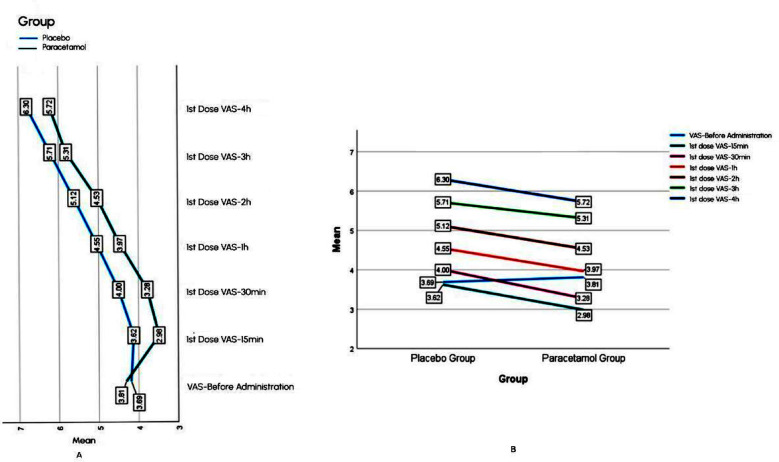
Comparison of within subject effect (A) and between subject analysis (B) of 1st dose.

**Fig.3:**
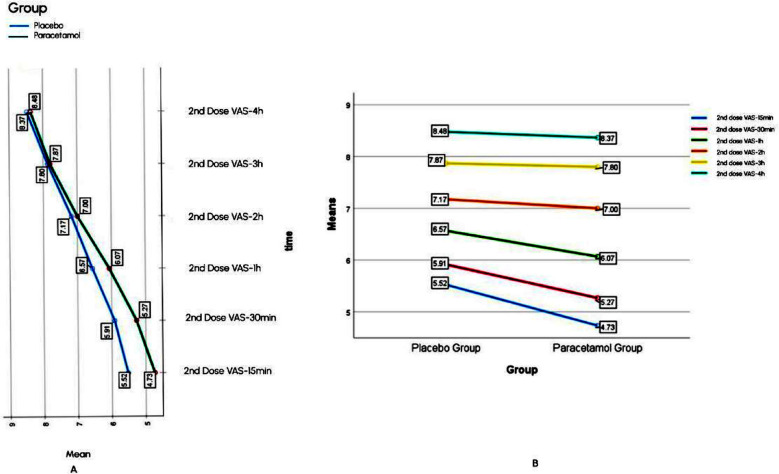
Comparison of within subject effect (A) and between subject analysis (B) of 2nd dose.

For first dose, Mean VAS in acetaminophen infusion group was lower than placebo group, at all measured time intervals, as mentioned in the Fig.2B. In acetaminophen infusion group it was seen that VAS first decreased as an immediate effect of drug later on it increased with time, but this increase was less as compared to placebo group (Fig.2A). Mean VAS for second dose in acetaminophen infusion group was lower than placebo till one hour post drug administration, after that VAS between groups was approximately equal at two, three and four hours (Fig.3B). Mean VAS within acetaminophen group and placebo Group-Increased with time (Fig.3A).

Statistical significance of observed mean VAS difference between subjects and within subjects (Fig. 2 and 3) was calculated by split plot ANOVA. Between subjects’ interaction for first dose was found significant with F value of 9.18, Mean square error of 5.89, and *p*-value of 0.003. For second dose this interaction resulted in non-significant *p*-value of 0.21 (F value=1.63, mean square error=6.98). Partial eta square showed 6.5% variance between subjects for first dose and 3.1% for second dose. Within subject interaction of time and group for first dose was significant with *p*-value of <0.001, F value of 622.63, and mean square error of 0.342. Similarly, this interaction for second dose was also significant with *p*-value of 0.005, F value=4.771, and mean square error=0.487.

## DISCUSSION

Evaluating analgesic efficacy of acetaminophen infusion in labour, for first dose, pain score at all intervals in acetaminophen infusion group was found significantly less than placebo. This finding positively indicated decreased pain intensity among parturients for four hours on the use of acetaminophen infusion in early part of active phase of first stage of labour.

The VAS score measured for the first dose was significantly less than placebo group at all measured intervals. For instance, at 15 minutes VAS was 2.98 with acetaminophen infusion vs 3.62 with placebo. In literature, some clinical trials designed similarly observed the same effect.^12^-^14^ A study reported that within 30 minutes after administration of acetaminophen infusion VAS in placebo group was 7.82 vs. 6.46 in acetaminophen infusion group, with *p*-value of <0.001.^12^ These aforementioned studies available in literature only observed the pain-relieving effect of acetaminophen infusion at 30 minutes or one hour interval after drug administration. We inquired the pain reduction due to studied drug from the start of active phase of first stage of labour (three cm cervical dilatation) till the beginning of second stage, for most women enrolled in this study. Pain intensity measured in this study at all intervals was observed to be less than the previously discussed studies. This indicated that the use of acetaminophen infusion in early phase of labour, decreased overall pain score at all intervals.

In this study we observed four hours analgesic effect of acetaminophen infusion with first dose. An observational study also provided supporting evidence but, it was designed to analyse pain score of acetaminophen infusion and tramadol in labour.^15^ In contrariety to above discussion a few studies in literature also reported analgesic efficacy of acetaminophen infusion for only two to three hour post administration, also concluding that acetaminophen infusion was observed to be less efficacious than opioids (pethidine, meperidine and nalbuphine).^16^-^18^ However, findings of aforementioned studies were opposed by different studies comparing acetaminophen infusion with above mentioned drugs separately.^19^,^20^ Some emphasized that analgesic efficacy of acetaminophen infusion was even better than opioid like tramadol and pethidine.^21^,^22^

For the second dose, we did not find significant decrease in pain score between groups. We can explain this finding as labour pain for first dose was predominantly originated in cervix and lower uterine segment and was visceral in nature. Near the end of first stage of labour the additional pain arose from the pressure caused by fetal descent on perineum resulting in both visceral and somatic pain.^23^ Labour pain at this point in time concluded to be unresponsive to analgesic effect of acetaminophen infusion. We did not find any study in literature comparing analgesic efficacy of acetaminophen infusion by repeating dose after four hour interval or designed to see efficacy in near second stage of labour.

To observe the pattern of pain scores in both groups with time, it was seen that in acetaminophen infusion group pain was found to decrease as an immediate effect of drug then it increased for both doses. As we know labour is an active process of childbirth. The first stage of labour is characterized by regular uterine contractions. These contractions increase in intensity and frequency as labour progresses towards second stage. Pain occurs due to these contractions causing stretch of cervix, ischemia of the muscle wall of uterus along with accumulation of lactate resulting in nerve fibers stimulation. Two randomized controlled trials, comparing analgesic efficacy of acetaminophen infusion with pethidine and nalbuphine supported this finding.^19^,^20^

### Limitations.

VAS, which was chosen as the main tool for pain assessment, is subjective in nature. This study also had small sample size especially for second dose as most of the women enrolled delivered before completion of eight hours interval.

### Suggestions:

We suggest studies must be done in our set-up to see adjunct analgesic effect of acetaminophen infusion with other analgesics especially opioids. More studies should be planned to determine acetaminophen infusion efficacy in primigravida. In prenatal check-ups, guidelines must be introduced in our country to help women understand the pharmacological and non-pharmacological methods of pain relief available to them and their mechanism of action. They should be allowed to make an informed decision regarding intrapartum analgesic use. Our findings implied that acetaminophen leads to a substantial reduction in pain perception during the active stage of labor. This provides evidence supporting the use of Acetaminophen as a standard analgesic in maternity units.

## CONCLUSION

The takeaway message of current study is that acetaminophen infusion is a potent labour analgesic with marked pain reduction in first stage of labour evident in the form of decreased VAS score. Its use will benefit primigravida in making labour less painful for them.

### Authors’ Contribution:

**WN:** Designed, registered trial in ICTR, helped in data collection, statistical analysis, data interpretation and manuscript writing.

**NK:** Did data collection, interpretation and Review of the manuscript.

**MN:** Conceived the idea, helped in designing, supervised the entire study, did editing of manuscript and is responsible for integrity of research.

**MAK:** Did statistical analysis, data interpretation, final review and manuscript editing.

**AC:** Helped in data collection, manuscript review and final approval of manuscript.
